# Bortezomib is an effective enhancer for chemical probe-dependent superoxide detection

**DOI:** 10.3389/fmed.2022.941180

**Published:** 2022-12-21

**Authors:** Misaki Matsumoto, Hikari Sawada, Kazumi Iwata, Masakazu Ibi, Nozomi Asaoka, Masato Katsuyama, Kaori Shintani-Ishida, Hiroshi Ikegaya, Shigehiko Takegami, Atsushi Umemura, Chihiro Yabe-Nishimura

**Affiliations:** ^1^Department of Pharmacology, Kyoto Prefectural University of Medicine, Kyoto, Japan; ^2^Radioisotope Center, Kyoto Prefectural University of Medicine, Kyoto, Japan; ^3^Department of Forensic Medicine, Kyoto Prefectural University of Medicine, Kyoto, Japan; ^4^Department of Analytical Chemistry, Kyoto Pharmaceutical University, Kyoto, Japan

**Keywords:** bortezomib, superoxide, NADPH oxidase, xanthine oxidase, peptide boronic acid

## Abstract

Various chemical probes for the detection of reactive oxygen species have been developed to examine oxidative stress associated with different pathologies. L-012, a luminol-based chemiluminescent probe, is widely used to detect extracellular superoxide because of its high sensitivity. We herein demonstrated that the co-application of the peptide boronic acid proteasome inhibitor, bortezomib, with L-012 significantly increased its luminescence without affecting the background. More than a 5-fold increase was detected in the total luminescence of L-012 in both NADPH oxidase-expressing cells and the xanthine oxidase-dependent cell-free superoxide generation system, but not in their background. Therefore, bortezomib increased the signal-to-background ratio and improved the detection of low levels of superoxide. The application of MLN2238, another peptide boronic acid proteasome inhibitor, also enhanced the luminescence of L-012. In contrast, carfilzomib, an epoxyketone proteasome inhibitor, did not increase luminescence, suggesting that the effects of bortezomib depend on the chemical structure of the peptide boronic acid, but not on its pharmacological effects. Bortezomib-induced enhancements appeared to be specific to the detection of superoxide because the detection of H_2_O_2_ by Amplex Red/HRP was not affected by the application of bortezomib. In the quantitative detection of the superoxide-specific oxidative product 2-hydroxyethidium (2-OH-E^+^), the application of bortezomib resulted in a 2-fold increase in the level of 2-OH-E^+^. Therefore, bortezomib sensitizes the detection of superoxide in both cell-based and cell-free systems, highlighting a novel feature of compounds containing the peptide boronic acid as powerful enhancers for the detection of superoxide.

## 1 Introduction

Reactive oxygen species (ROS) exert pleiotropic effects, such as host defenses, cell proliferation, cellular senescence, cell death, and disease progression (1). ROS, including superoxide (O_2_⋅^–^), hydrogen peroxide (H_2_O_2_), the hydroxyl radical, hypochlorous acid, and peroxynitrite (ONOO^–^), are highly reactive and short-lived; therefore, chemical probes, such as luminol, L-012, H_2_DCFDA, and hydroethidine (HE, also known as dihydroethidium), are used to detect these species. Although the detection of intracellular ROS is relatively challenging due to the presence of various oxidants, such as heme proteins, cytochrome *c*, iron, and peroxidase, in cells (2), the detection of extracellular ROS is relatively reliable due to the absence of these contaminants. The neutrophil oxidative burst, the typical generation of extracellular O_2_⋅^–^ by NOX2/NADPH oxidase, is easily detected using chemical probes (3). Among the various chemical probes used to detect ROS, L-012, a luminol-based chemiluminescent probe (4), is widely used for the real-time monitoring of extracellular O_2_⋅^–^ due to its sensitivity (5–8). L-012 may also be employed for the *in vivo* detection of O_2_⋅^–^ (9, 10). However, in many cells in which the endogenous expression of NADPH oxidase is very low, difficulties are associated with detecting ROS due to the low output of O_2_⋅^–^.

By using L-012 to screen for potential inhibitors of NADPH oxidase, we unexpectedly found that bortezomib, a peptide boronic acid proteasome inhibitor, significantly increased luminescence without affecting the background. We herein demonstrated that the enhancement induced by bortezomib was specific to the O_2_⋅^–^ detection system and dependent upon the chemical structure of the peptide boronic acid. The present results suggest the potential of bortezomib as an effective enhancer in the O_2_⋅^–^ detection system.

## 2 Materials and methods

### 2.1 Plasmids, cells, and cell culture

Plasmid constructs encoding human NOX1, NOXA1, and NOXO1 were previously established (11). HEK293 cells and RAW264.7 cells were obtained from ATCC, and maintained in Dulbecco’s modified Eagle’s medium containing 10% fetal bovine serum, 100 units/ml penicillin, and 100 μg/ml streptomycin at 37^°^C under an atmosphere of 95% air and 5% CO_2_. To measure the production of O_2_⋅^–^, the NOX1, NOXA1, and NOXO1 plasmids were cotransfected into HEK293 cells using ScreenFect™ A (Wako, Osaka, Japan) and analyzed 24–48 h after transfection.

### 2.2 Measurement of O_2_⋅^–^ production using L-012

As previously described (7), NOX1-transfected cells or RAW264.7 cells were suspended in Krebs-HEPES buffer and then transferred to a 96-well white plate on ice at a density of 1 × 10^5^ cells/well or 1 × 10^4^ cells/well, respectively. Cells were pre-incubated with DMSO/bortezomib (Wako) at a volume of 90 μl on ice for 10 min. In some experiments, carfilzomib (Adipogen Life Sciences, CA, USA) or MLN2238 (Cayman Chemical, MI, USA) was used. Immediately after the addition of 10 μl of L-012 (final concentration of 100 μM, Wako), chemiluminescence was measured at 37^°^C for 30 min using a luminescent microplate reader (Centro LB960, Berthold, Bad Wildbad, Germany). Phorbol 12-myristate 12-acetate (PMA, 200 nM), which activates protein kinase C, was added with L-012 to RAW267.4 cells in order to induce an oxidative burst. The chemiluminescence of L-012 was expressed as relative luminescence units (RLU) and measured every 1 min. Superoxide dismutase (SOD, 5 U/ml, sigma) was pre-incubated with cells or the xanthine-xanthine oxidase (X/XO) system for 10 min on ice and L-012-dependent luminescence was then measured.

### 2.3 O_2_⋅^–^ generation in the X/XO system

Xanthine oxidase (XO) (Wako, final 5 mU/ml) dissolved in Krebs-HEPES buffer was pre-incubated with DMSO/bortezomib at a volume of 80 μ l in a 96-well white plate on ice for 10 min. Immediately after the addition of 10 μ l of L-012 (final concentration of 100 μ M) and 10 μ l of xanthine (final concentration of 0.2 mM, Nacalai Tesque, Japan), chemiluminescence was measured at 37^°^C for 30 min.

### 2.4 Measurement of the O_2_⋅^–^-specific oxidation product 2-OH-E^+^ by LC-MS/MS

As previously described (12, 13), cells or the X/XO system was pre-incubated with Krebs-HEPES buffer containing freshly prepared 10 μM HE (Cayman Chemical), the chelating agent diethylenetriaminepentaacetic acid (0.1 mM, Sigma), and DMSO/bortezomib at 37^°^C for 60 min. Three hundred microliters of the supernatant fraction was mixed with an equal amount of ice-cold acetonitrile containing 0.1% formic acid and 1 μM of 3,8-diamino-6-phenylphenanthridine (DAPP) as an internal control. After centrifugation at 10,000 × *g* at 4^°^C for 5 min, the supernatant was analyzed using the LC-MS/MS system (LCMS-8045, Shimadzu, Kyoto, Japan). Analytes were detected using the multiple-reaction monitoring mode with the following transitions: 316.30 > 210.10 (HE), 329.90 > 300.00 (2-OH-E^+^), 314.30 > 284.05 (E^+^), and 286.25 > 208.10 (DAPP). The contents of HE, 2-OH-E^+^, and ethidium (E^+^) were expressed as a peak area ratio to DAPP.

### 2.5 Measurement of H_2_O_2_ production using Amplex Red/HRP

Cells or the X/XO system was pre-incubated with DMSO/bortezomib at a volume of 50 μl of Krebs-HEPES buffer for 10 min in a 96-well white plate on ice. After the addition of 50 μl of reaction buffer containing Amplex Red (final concentration of 50 μM, Thermo Fisher Scientific, USA) and horseradish peroxidase (HRP, final concentration of 0.1 U/ml, Merck, Darmstadt, Germany), the microplate was incubated at 37^°^C for 30 min. Fluorescence (Ex. 540 nm/Em. 590 nm) was measured using a microplate reader (Spectra Max^®^ M2, Molecular Devices, USA) and expressed as relative fluorescence units.

### 2.6 Measurement of O_2_⋅^–^ production using the cytochrome c reduction assay

As previously described (3, 14), cells or the X/XO mixture were pre-incubated with DMSO/bortezomib at a volume of 90 μl of Krebs-HEPES buffer containing 0.1 mM EDTA for 10 min in a 96-well plate on ice. To evaluate the superoxide-dependent reaction, some reactions were performed in the presence of SOD (5 U/ml). After the addition of 10 μl of cytochrome *c* (1 mM, Nacalai Tesque, Japan), absorbance at 550 and 670 nm (reference) was continuously monitored at 37^°^C for 30 min using a microplate reader kinetic mode (Spectra Max^®^ M2). The optical density (OD) in the presence of SOD was subtracted from each of reaction to show SOD-inhibitable cytochrome *c* reduction. The rate of O_2_⋅^–^ production was calculated from the linear part of the graph using an extinction coefficient of 21.1 mM^–1^ cm^–1^.

### 2.7 Statistical analysis

Results are expressed as the mean ± standard error of the mean (SEM). Statistical analyses were performed using a one-way analysis of variance followed by the *post hoc* Tukey-Kramer test.

## 3 Results

### 3.1 Bortezomib enhanced the luminescence of L-012 in O_2_⋅^–^ production systems

**FIGURE 1:**
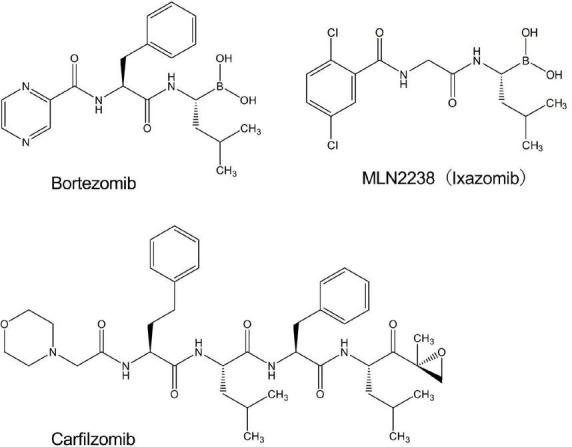
Chemical structures of drugs used in the present study.

**FIGURE 2:**
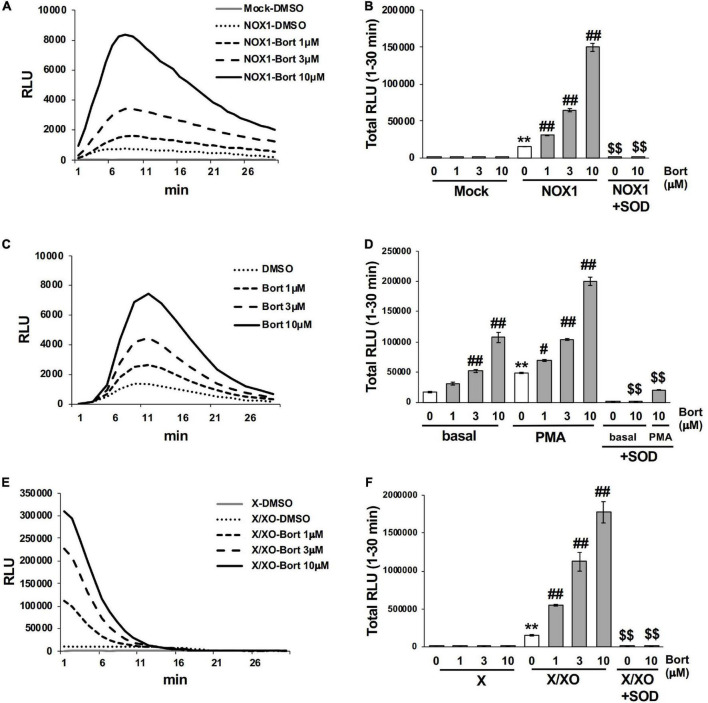
Bortezomib enhanced the luminescence of L-012 in superoxide production systems. **(A)** Time course of L-012 luminescence in HEK293 cells overexpressing human NOX1/NADPH oxidase (NOX1, 1 × 10^5^ cells/0.1 ml). The chemiluminescence of L-012 was expressed as relative luminescence units (RLU). **(B)** Total counts of L-012 luminescence during the 30 min measurement. Superoxide dismutase (SOD, 5 U/ml) was pre-incubated with bortezomib/DMSO for 10 min before measurements. Time course **(C)** and total counts **(D)** of L-012 luminescence in RAW264.7 (1 × 10^4^ cells/0.1 ml). PMA (200 nM) was added to induce an oxidative burst in **(D)**. Time course **(E)** and total counts **(F)** of L-012 luminescence in the cell-free superoxide production system. X (0.2 mM) and XO (5 mU/ml) were used. *N* = 3. Data were from three independent experiments and expressed as the mean ± SEM. **P < 0.01 versus the control (Mock, basal, or X). ^#^<0.05 and ^##^*P* < 0.01 versus 0 μM. ^$$^*P* < 0.01 versus the control without SOD.

We performed screening using a library containing more than 3,000 chemical compounds to identify potential inhibitors of NADPH oxidase with the chemiluminescent probe L-012. In this process, we unexpectedly found that bortezomib (Figure 1), a peptide boronic acid proteasome inhibitor, markedly increased the luminescence of L-012. In cells expressing human NOX1/NADPH oxidase (1 × 10^5^ cells/0.1 ml), L-012 luminescence was dose-dependently increased by the application of bortezomib (Figures 2A,B). Bortezomib did not amplify the luminescence of L-012 in mock-transfected cells that did not produce O_2_⋅^–^ (Figure 2B). Furthermore, a cotreatment with SOD completely attenuated luminescence, suggesting that the L-012-dependent signal was attributed to the production of O_2_⋅^–^ (Figure 2B).

The effects of bortezomib on the oxidative burst were examined in phagocytic cells. The mouse macrophage cell line RAW264.7 (1 × 10^4^ cells/0.1 ml), which endogenously expresses NOX2/NADPH oxidase (15), produced an equivalent level of O_2_⋅^–^ to 1 × 10^5^ NOX1-overexpressing cells, under basal conditions (Figure 2C). A significant level of O_2_⋅^–^ was detected by L-012 chemiluminescence following the stimulation with PMA (Figure 2D). Bortezomib dose-dependently enhanced luminescence, which was suppressed by the application of SOD (Figures 2C,D). At the higher luminescence signals produced by 1 × 10^5^ RAW264.7 cells, the enhancement induced by bortezomib was less noticeable possibly due to the saturation of the signals (data not shown).

Similarly, bortezomib-induced enhancements in chemiluminescence were observed in the cell-free system using X/XO. The addition of the substrate (X) to XO (5 mU/ml) immediately initiated O_2_⋅^–^ production, as shown in Figure 2E. The rapid decrease in L-012 luminescence after 15 min appears to be attributed to inactivation of XO by H_2_O_2_ generated during X/XO reaction (16), since the addition of fresh XO at 30 min greatly improved luminescence (data not shown). Bortezomib dose-dependently enhanced luminescence without any effects on the XO-free negative control (Figures 2E,F). Luminescence was again sensitive to SOD (Figure 2F). Simultaneously, the X/XO reaction yields uric acid as a reaction product; however, bortezomib did not affect the uric acid production (Supplementary Figure 1). Thus, bortezomib sensitized the detection of O_2_⋅^–^ without affecting the enzyme activity of XO. Overall, the application of bortezomib with L-012 significantly enhanced luminescence without affecting the background in both the cell-based and cell-free systems of O_2_⋅^–^ production.

When higher concentrations of bortezomib (30 or 100 μM) were applied, bortezomib enhanced the luminescence of L-012 in NOX1-overexpressing cells and the X/XO system (Supplementary Figure 2). However, the higher concentration of bortezomib increased the luminescence of L-012 in the XO-free system (negative control) for an unknown reason (Supplementary Figure 2B). Regarding cell toxicity, a 60 min incubation with bortezomib in NOX1-expressing cells did not deplete ATP, an indicator of the loss of viable cells, even at 100 μM (Supplementary Figure 3). Based on these results, we applied 10 μM of bortezomib, which does not affect the background, in subsequent experiments.

### 3.2 Bortezomib improved the detection of lower levels of O_2_⋅^–^

**FIGURE 3:**
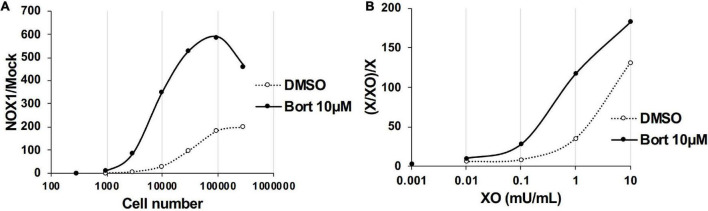
Bortezomib improved the detection of lower levels of superoxide. **(A)** The ratio of total L-012 luminescence during 30 min in 300 - 3 × 10^5^ NOX1-expressing cells to that in mock-transfected cells. **(B)** The ratio of total L-012 luminescence during 30 min in the X/XO system (0.001 – 10 mU/ml) to that in xanthine-containing medium (X, 0.2 mM). The total volume of each experiment was 0.1 ml. Representative data of two independent experiments are shown.

We examined the effects of bortezomib on the detection of lower levels of O_2_⋅^–^ using a smaller number of NOX1-overexpressing cells (<1 × 10^5^) or lower concentration of XO (<5 mU/ml). In the absence of bortezomib, more than 1 × 10^4^ cells overexpressing NOX1 or 1 mU/ml XO was required to clearly differentiate luminescence from the background (Figure 3, See the DMSO control). On the other hand, only ∼3,000 NOX1-overexpressing cells or 0.1 mU/ml of XO was required for the L-012-dependent detection of O_2_⋅^–^ in the presence of bortezomib (Figure 3). Therefore, the application of bortezomib appeared to be useful for detecting lower levels of O_2_⋅^–^.

### 3.3 Bortezomib-induced enhancements in L-012 luminescence were superior to HRP-induced amplification

In previous studies, L-012 and isoluminol were used in combination with HRP to enhance their luminescence (3, 17). As shown in Table 1, the co-application of 0.1 U/ml HRP with L-012 resulted in an approximately 100-fold increase in the total luminescence of L-012 in NOX1-expressing cells. However, it also induced an approximately 200-fold increase in the luminescence of L-012 in mock-transfected cells. The signal-to-background ratio (S/B) decreased from 201.4 to 80.4 (Table 1). On the other hand, the application of bortezomib with L-012 increased S/B to 555.9 because it did not affect luminescence in mock-transfected cells (Table 1). Similar results were obtained in the X/XO system (Table 2). These results demonstrated that the application of bortezomib was superior to the existing HRP-induced L-012 amplification method.

**TABLE 1:** Effects of bortezomib (Bort, 10 μM) or HRP (0.1 U/ml) on L-012 luminescence in NOX1-expressing cells (1 × 10^5^ cells/0.1 ml).

	DMSO	Bort	HRP
Mock	226 ± 3	267 ± 20	55,743 ± 732
NOX1	45,460 ± 189	148,428 ± 12,492	4,481,212 ± 27,469
NOX1/Mock	201.4	555.9	80.4

The total counts of L-012 luminescence during 30 min and the ratio (NOX1/Mock), corresponding to S/N, are shown. *N* = 3. Data were from two independent experiments, and expressed as the mean ± SEM.

**TABLE 2:** Effects of bortezomib (Bort, 10 μM) or HRP (0.1 U/ml) on L-012 luminescence in the X/XO system.

	DMSO	Bort	HRP
X	2,149 ± 229	3,419 ± 305	416,968 ± 109,594
X/XO	83,877 ± 5,411	1,385,547 ± 14,406	8,378,394 ± 217,303
(X/XO)/X	39.0	405.2	20.1

The total counts of L-012 luminescence during 30 min and the ratio [(X/XO)/X], corresponding to S/N, are shown. *N* = 3. Data were from two independent experiments, and expressed as the mean ± SEM.

### 3.4 Bortezomib-induced enhancements in L-012 luminescence were dependent on the chemical structure of the peptide boronic acid and specific to the detection of O_2_⋅^–^

**FIGURE 4:**
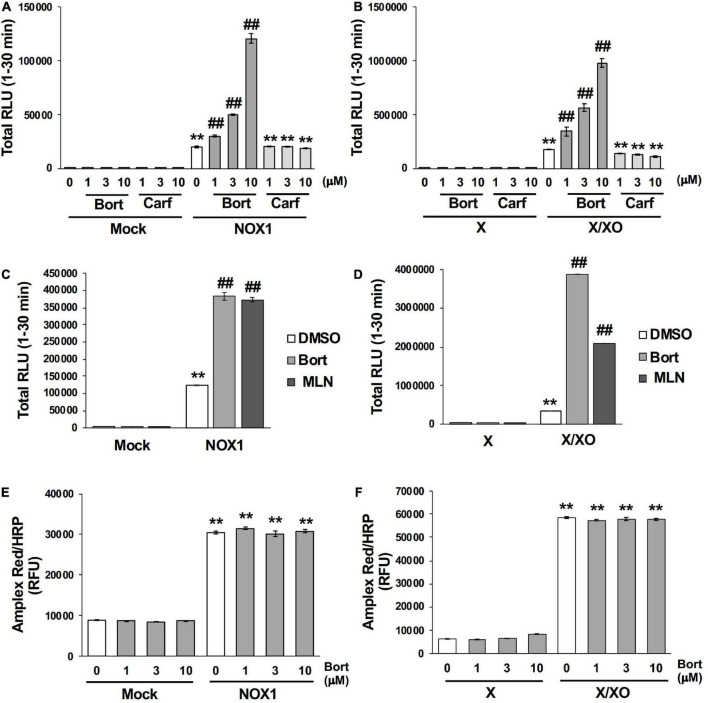
Bortezomib-induced enhancements in L-012 luminescence were dependent on chemical structure of the peptide boronic acid and specific to the detection of superoxide. Effects of bortezomib (Bort) or carfilzomib (Carf) on total L-012 luminescence during the 30 min measurement in NOX1-expressing cells [**(A)**, 1 × 10^5^ cells/0.1 ml] and the X/XO system [**(B)**, 0.2 mM X and 5 mU/ml XO]. The effects of bortezomib or MLN2238 (MLN, 10 μM) on L-012 luminescence in NOX1-expressing cells **(C)** and the X/XO system **(D)**. Effects of bortezomib on the Amplex Red/HRP-dependent detection of H_2_O_2_ in NOX1-expressing cells **(E)** and the X/XO system **(F)**. The fluorescence of Amplex Red/HRP was expressed as relative fluorescence units (RFU). *N* = 3. Data were from two independent experiments, and expressed as the mean ± SEM. **P < 0.01 versus the control (Mock or X). ^##^*P* < 0.01 versus 0 μM.

Bortezomib is a peptide boronic acid proteasome inhibitor that is used to treat multiple myeloma (18). We herein examined carfilzomib (Figure 1), an epoxyketone proteasome inhibitor that is also used to treat multiple myeloma (18, 19). As shown in Figures 4A,B, carfilzomib did not affect L-012 luminescence in NOX1-expressing cells or in the X/XO system. In contrast, MLN2238 (Figure 1), another type of peptide boronic acid proteasome inhibitor, also enhanced L-012 chemiluminescence (Figures 4C,D). On the other hand, 17 chemicals containing phenyl boronic acid did not affect the luminescence of L-012 (Supplementary Figure 4), suggesting that the chemical structure of the peptide boronic acid, but not boronic acid itself, is critical for enhancements in the luminescence of L-012.

The effects of bortezomib on the detection of H_2_O_2_ were assessed using the Amplex Red/HRP system. A large amount of fluorescence was detected in NOX1-overexpressing cells as well as in the X/XO system after a 30 min incubation with the Amplex Red probe in the presence of HRP at 37^°^C (Figures 4E,F). However, the co-application of bortezomib with Amplex Red/HRP did not affect the total fluorescent signal (Figures 4E,F). Furthermore, bortezomib did not affect the X/XO-dependent luminescence in the ROS-glo™ H_2_O_2_ assay (Promega, USA) which does not require HRP (Supplementary Figure 5). These results indicated that enhancements in L-012 luminescence by bortezomib were specific to the detection of O_2_⋅^–^, but not H_2_O_2_.

### 3.5 Bortezomib increased the level of the O_2_⋅^–^-specific oxidative product 2-OH-E^+^

**FIGURE 5:**
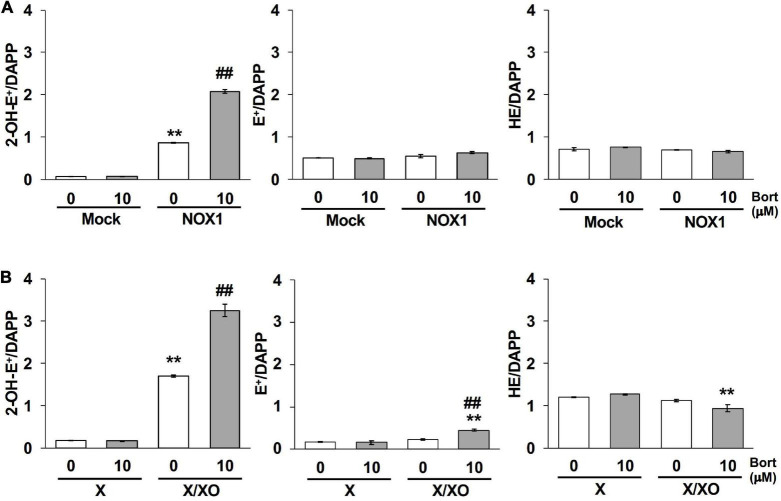
Bortezomib increased the level of the superoxide-specific oxidative product 2-OH-E^+^. The levels of 2-OH-E^+^, E^+^, and HE in the supernatant of NOX1-expressing cells [**(A)**, 2 × 10^6^ cells/mL] and the X/XO system [**(B)**, 0.2 mM X and 5 mU/ml XO] measured by LC-MS/MS. Data were expressed as a ratio to DAPP, an internal control. *N* = 3. Data were from two independent experiments, and expressed as the mean ± SEM. **P < 0.01 versus the control (Mock or X). ^##^*P* < 0.01 versus 0 μM.

The quantification of 2-OH-E^+^, a O_2_⋅^–^-specific oxidative product of the HE probe (12, 13), was performed using LC-MS/MS to verify the effects of bortezomib on a different O_2_⋅^–^ detection system. As shown in Figure 5A, 2-OH-E^+^ was clearly detected in the supernatant of NOX1-expressing cells, but was negligible in mock-transfected cells after a 60 min incubation with the HE probe at 37^°^C. Neither the level of E^+^, a non-specific oxidative metabolite of HE, nor that of HE itself was affected by the overexpression of NOX1 (Figure 5A). The co-incubation of bortezomib with the HE probe induced a 2-fold increase in the level of 2-OH-E^+^ in NOX1-expressing cells, but did not change the level of E^+^ or HE (Figure 5A). Bortezomib did not affect the level of HE-related products in mock-transfected cells (Figure 5A). Similarly, a high level of 2-OH-E^+^ was detected in the X/XO reaction mixture, with a 2-fold increase being observed in the presence of bortezomib (Figure 5B). The level of E^+^ in the X/XO mixture slightly increased in the presence of bortezomib. In contrast, bortezomib significantly decreased the level of HE in the X/XO supernatant, suggesting the increased consumption of HE by O_2_⋅^–^. Therefore, the application of bortezomib markedly improved the detection of O_2_⋅^–^ by the HE- and L-012-based systems.

### 3.6 Bortezomib-induced enhancement was not observed in the cytochrome c reduction assay

**FIGURE 6:**
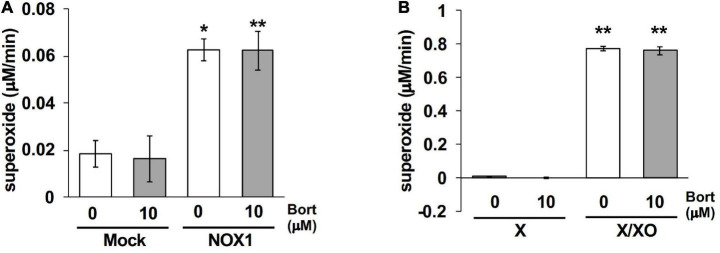
Bortezomib-induced enhancement was not observed in the cytochrome *c* reduction assay. The levels of SOD-inhibitable reduced cytochrome *c* in the supernatant of NOX1-expressing cells [**(A)**, 1 × 10^5^ cells/0.1 ml] and of the X/XO system [**(B)**, 5 mU/ml XO]. The concentration of cytochrome *c* and X was 0.1 and 0.2 mM, respectively. The absorbance was measured at 30 min after the 37^°^C reaction and expressed as optical density at a wavelength of 550 nm and a reference wavelength of 670 nm. *N* = 3. Data were from two independent experiments, and expressed as the mean ± SEM. *P < 0.05 and **P < 0.01 versus the control (Mock or X).

Finally, effects of bortezomib on the cytochrome *c* reduction assay, a classical assay to detect O_2_⋅^–^, were examined. Cytochrome *c* is a 12-kDa heme-containing protein that can be reduced by O_2_⋅^–^, exhibiting an absorbance at 550 nm. To detect superoxide-specific cytochrome *c* reduction, the SOD-inhibitable reaction was measured. As the cytochrome *c* reduction assay is less sensitive compared to other methods (3), NOX1-expressing cells showed a small increase in the level of reduced cytochrome *c* (0.06 μM/min, Figure 6A). The application of bortezomib did not further increase its level (Figure 6A). Similarly, bortezomib-induced enhancement was not observed in the cytochrome *c* reduction assay using X/XO system (0.77 μM/min, Figure 6B). These data indicate that the bortezomib-induced enhancement in O_2_⋅^–^ detection is not versatile for all O_2_⋅^–^ detection systems, but demonstrates different compatibility with probes. Furthermore, these results suggest that bortezomib does not increase O_2_⋅^–^ flux, but sensitizes the chemical probe-dependent O_2_⋅^–^ detection.

## 4 Discussion

Screening for chemical compounds that inhibit the activity of NADPH oxidase revealed the unique characteristic of bortezomib to enhance the detection of O_2_⋅^–^. Since experimental measurements of lower levels of O_2_⋅^–^ have been highly challenging, the present results may be useful for improving existing methods to detect O_2_⋅^–^ in cell-based and cell-free systems.

Bortezomib-induced enhancements in L-012 luminescence appeared to be dependent on the chemical structure of the peptide boronic acid because luminescence increased in the presence of MLN2238, another peptide boronic acid proteasome inhibitor. On the other hand, neither carfilzomib, an epoxyketone proteasome inhibitor, nor chemicals containing phenyl boronic acid affected the luminescence of L-012. Bortezomib enhanced the detection of O_2_⋅^–^ in a cell-free system, which provides further support for proteasome inhibition by bortezomib being unrelated to enhancements in L-012 luminescence. Chemicals containing phenyl boronic acid, such as coumarin boronic acid (CBA), have recently been used as fluorescent probes for the detection of H_2_O_2_ and ONOO^–^ (20, 21). Since bortezomib did not alter the detection of H_2_O_2_ by Amplex Red/HRP and chemicals containing phenyl boronic acid did not affect L-012 luminescence (Supplementary Figure 4), the effects of bortezomib on O_2_⋅^–^ appear to differ from those of CBA. Although the boronic acid group acts as an electron-withdrawing group (22), the structural difference adjacent to boronic acid motif may contribute to their divergent characteristics.

Enhancements in the detection of O_2_⋅^–^ by bortezomib may be useful for the assessment of O_2_⋅^–^ production in both cell-based and cell-free systems, particularly under conditions of low-output states in which signals are very weak. The present results revealed the prominent effects of bortezomib for the detection of O_2_⋅^–^ generated in a small number of NOX1-expressing cells as well as in a cell-free assay with a limited amount of XO (0.1 mU/ml, the estimated superoxide flux is 0.015 μM/min). On the other hand, at a higher level of O_2_⋅^–^, bortezomib-induced enhancements became less noticeable (Figure 3). The lack of toxicity of bortezomib in the assay was supported by ATP not being depleted following the treatment at concentrations as high as 100 μM for 60 min (Supplementary Figure 3). However, since the IC_50_ of bortezomib against proteasomes is 2.4 nM (19), a prolonged incubation needs to be avoided in cell-based experiments. Therefore, the use of bortezomib for the *in vivo* detection of L-012-dependent O_2_⋅^–^ may be limited because a relatively high dose of bortezomib is required to enhance the signal.

It is important to note that bortezomib did not affect the background, which markedly improved S/B. In contrast, the detection of O_2_⋅^–^ using L-012 with HRP (17) increased both the signal and background, thereby decreasing S/B (Tables 1, 2). Furthermore, the use of the L-012 probe in combination with HRP is associated the following issue: L-012 is oxidized to the L-012 radical *via* one-electron oxidation in the presence of HRP, which reacts with oxygen to generate O_2_⋅^–^ (i.e., redox cycling) (23). Therefore, the increased background signals observed in the presence of HRP in this study may be attributed to redox cycling in the reaction. Accordingly, the present assay using L-012 with bortezomib is superior to the existing method using L-012 with HRP.

The extent of bortezomib-induced amplification differs between O_2_⋅^–^ detection methods. The amplification of L-012 luminescence by bortezomib was more than 5-fold (Figure 2), while that of 2-OH-E^+^ generation was 2-fold (Figure 5). On the other hand, bortezomib did not amplify the level of reduced cytochrome *c* (Figure 6). The lack of enhancement by bortezomib in the cytochrome *c* reduction assay suggests that bortezomib does not increase O_2_⋅^–^ flux but sensitizes the chemical probe-dependent O_2_⋅^–^ detection, possibly by catalyzing an electron transfer reaction between O_2_⋅^–^ and chemical probes with different compatibility. The lack of enhancement by bortezomib in H_2_O_2_-dependent Amplex Red/HRP assay also supports the notion that bortezomib does not increase O_2_⋅^–^ flux, since the generation of H_2_O_2_ is mainly attributed to the disproportionation of O_2_⋅^–^. As cytochrome *c* is a protein molecule in contrast to L-012 and HE, there may be a difference in compatibility with bortezomib. Further studies are needed to clarify the exact mechanisms by which bortezomib enhances the chemical probe-dependent O_2_⋅^–^ detection. Although the bortezomib-induced enhancement was not versatile for all O_2_⋅^–^ detection systems, the good compatibility of bortezomib with both L-012 and HE is powerful in improving the current method of O_2_⋅^–^ detection.

In conclusion, we identified a novel characteristic of bortezomib that sensitizes the chemical probe-dependent detection of O_2_⋅^–^. The chemical structure of the peptide boronic acid, but not that of the boronic acid itself, was critical for this effect. The simple application of bortezomib with O_2_⋅^–^-sensitive probes significantly enhanced low-output O_2_⋅^–^, thereby enhancing its detection. The present study may lead to the development of a more effective enhancer for the measurement of O_2_⋅^–^ levels by modifying the chemical structure of bortezomib. The assessment of oxidative stress in limited amounts of samples may become useful in future diagnostic applications.

## Data availability statement

The raw data supporting the conclusions of this article will be made available by the authors, without undue reservation.

## Author contributions

MM, HS, NA, KI, MI, and MK performed experiments. KS-I and HI performed LC-MS/MS. MM, ST, AU, and CY-N wrote the manuscript and prepared the figures. MM and CY-N designed the experiments. All authors contributed to the article and approved the submitted version.
