# Supplementary material for: Bortezomib is an effective enhancer for chemical probe-dependent superoxide detection
Source: Front Med (Lausanne). 2022 Dec 21;9:941180. doi: 10.3389/fmed.2022.941180 (PMC9811382; doi:10.3389/fmed.2022.941180)
Supplement: Supplementary file 1 [file Data_Sheet_1.pdf]

Supplementary Material

Supplemental figure 1

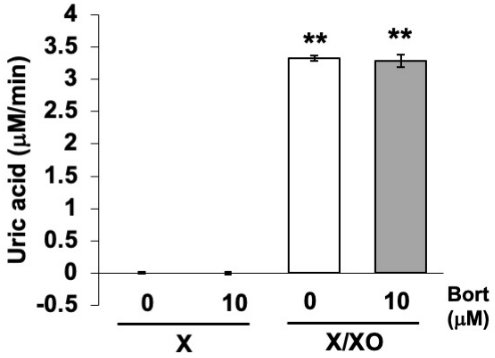

Supplemental fig. 1. Levels of uric acid production in the X/XO system. The absorbance at 292 nm for uric acid was measured during the measurement of 30 min at 37 °C, and the rate of uric acid production from the linear part of the graph was calculated using an extinction coefficient of 12.5 mM^{-1} cm^{-1}. N = 6. Data were from two independent experiments, and expressed as the mean ± SEM. $**P$ <0.01 versus the control (X).

Supplemental figure 2

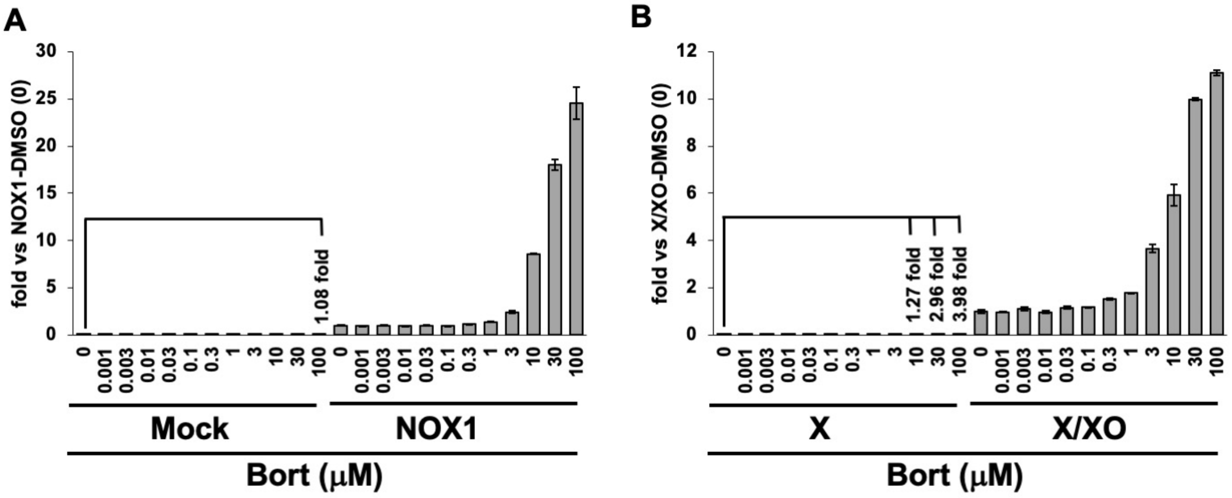

Supplemental fig. 2. Effects of higher concentrations of bortezomib (Bort, 0.001 – 100 μM) on total L-012 luminescence during the 30-min measurement in NOX1-expressing cells (A, 1×10^5 cells) and the X/XO system (B, 5 mU/mL XO). N = 3. Data were from two independent experiments, and expressed as the mean ± SEM.

Supplemental figure 3

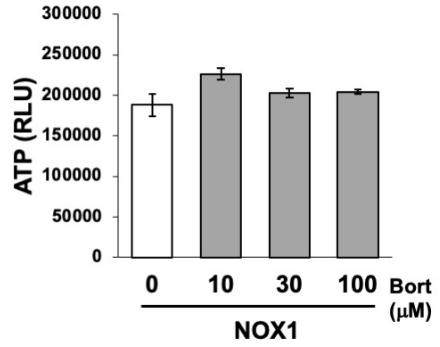

Supplemental fig. 3. Cell viability in the presence of bortezomib. Effects of bortezomib (Bort, 10 – 100 μM) on the viability of NOX1-expressing cells (1×10^5 cells) 60 min after an incubation at 37°C. Cell viability was measured using the CellTiter-Glo® luminescent cell viability assay (Promega, USA) and cellular ATP contents were expressed as relative luminescence units (RLU). N = 3. Data were from two independent experiments, and expressed as the mean ± SEM.

Supplemental figure 4

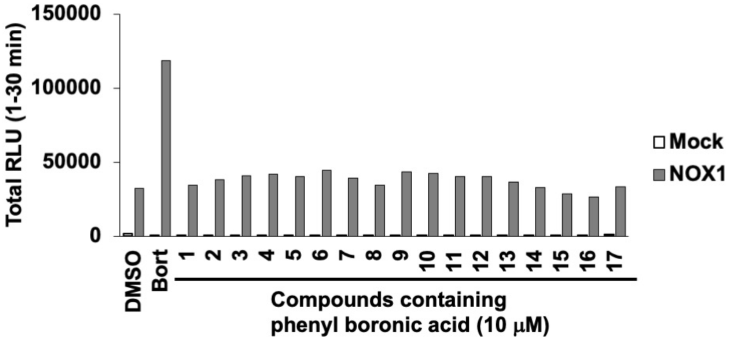

Compound 1

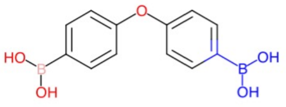

Compound 2

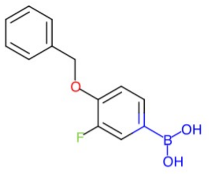

Compound 3

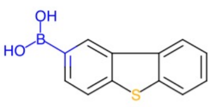

Compound 4

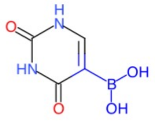

Compound 5

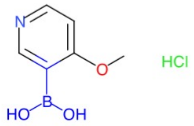

Compound 6

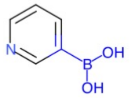

Compound 7

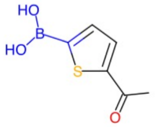

Compound 8

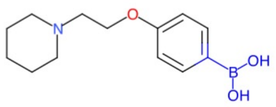

Compound 9

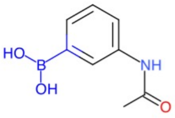

Compound 10

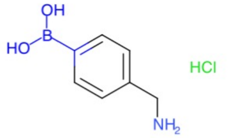

Compound 11

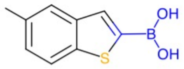

Compound 12

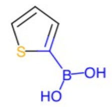

Compound 13

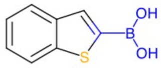

Compound 14

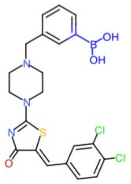

Compound 15

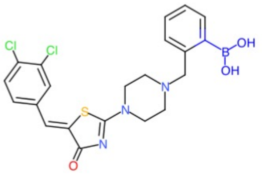

Compound 16

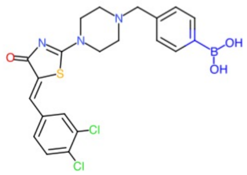

Compound 17

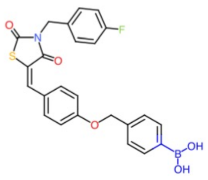

Supplemental fig. 4. Effects of compounds containing phenyl boronic acid on L-012 luminescence. Seventeen compounds containing phenyl boronic acid (1 – 17, 10 μM) or bortezomib (Bort, 10 μM) were pre-incubated with NOX1-expressing cells (1×10^5 cells) on ice for 10 min before the addition of L-012. The total counts of L-012 luminescence during 30 min were expressed.

Supplemental figure 5

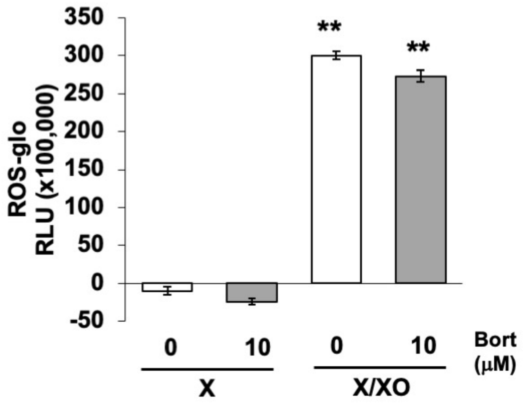

Supplemental fig. 5. Effects of bortezomib on the ROS-glo™ H_2O_2 assay (Promega, USA) which does not require HRP. According to the manufacturer's protocol, the X/XO system (0.2 mM X and 5 mU/mL XO) was incubated for 30 min at 37 °C with H_2O_2 Substrate Solution, followed by the addition of ROS-glo™ Detection Solution, then luminescence was measured. N = 3. Data were from two independent experiments, and expressed as the mean ± SEM.
